# Data infrastructures for AI in medical imaging: a report on the experiences of five EU projects

**DOI:** 10.1186/s41747-023-00336-x

**Published:** 2023-05-08

**Authors:** Haridimos Kondylakis, Varvara Kalokyri, Stelios Sfakianakis, Kostas Marias, Manolis Tsiknakis, Ana Jimenez-Pastor, Eduardo Camacho-Ramos, Ignacio Blanquer, J. Damian Segrelles, Sergio López-Huguet, Caroline Barelle, Magdalena Kogut-Czarkowska, Gianna Tsakou, Nikolaos Siopis, Zisis Sakellariou, Paschalis Bizopoulos, Vicky Drossou, Antonios Lalas, Konstantinos Votis, Pedro Mallol, Luis Marti-Bonmati, Leonor Cerdá Alberich, Karine Seymour, Samuel Boucher, Esther Ciarrocchi, Lauren Fromont, Jordi Rambla, Alexander Harms, Andrea Gutierrez, Martijn P. A. Starmans, Fred Prior, Josep Ll. Gelpi, Karim Lekadir

**Affiliations:** 1 FORTH-ICS, FORTH-ICS, N. Plastira 100, Heraklion, Crete Greece; 2 Quibim SL, Valencia, Spain; 3 Universitat Politècnica de València, Valencia, Spain; 4 European Dynamics, Maroussi, Athens Greece; 5 Timelex CVBA, Brussels, Belgium; 6 MAGGIOLI S.P.A., Research and Development Lab, Marousi, Greece; 7 Centre of Research & Technology - Hellas, Information Technologies Institute, Thermi - Thessaloniki, Greece; 8 La Fe Health Research Institute, Valencia, Spain; 9 Medexprim, Labège, France; 10 Department of Physics, University of Pisa, Pisa, Italy; 11 European Genome-Phenome Archive, Centre for Genomic Regulation, Barcelona, Spain; 12 Erasmus MC, Rotterdam, The Netherlands; 13 Department of Biomedical Informatics, University of Arkansas for Medical Sciences, Little Rock, AR USA; 14 University of Barcelona, Barcelona, Spain

**Keywords:** Artificial intelligence, Data anonymization, Data management, Diagnostic imaging, Neoplasms

## Abstract

Artificial intelligence (AI) is transforming the field of medical imaging and has the potential to bring medicine from the era of ‘sick-care’ to the era of healthcare and prevention. The development of AI requires access to large, complete, and harmonized real-world datasets, representative of the population, and disease diversity. However, to date, efforts are fragmented, based on single–institution, size-limited, and annotation-limited datasets. Available public datasets (*e.g.*, The Cancer Imaging Archive, TCIA, USA) are limited in scope, making model generalizability really difficult. In this direction, five European Union projects are currently working on the development of big data infrastructures that will enable European, ethically and General Data Protection Regulation-compliant, quality-controlled, cancer-related, medical imaging platforms, in which both large-scale data and AI algorithms will coexist. The vision is to create sustainable AI cloud-based platforms for the development, implementation, verification, and validation of trustable, usable, and reliable AI models for addressing specific unmet needs regarding cancer care provision. In this paper, we present an overview of the development efforts highlighting challenges and approaches selected providing valuable feedback to future attempts in the area.

**Key points**

• Artificial intelligence models for health imaging require access to large amounts of harmonized imaging data and metadata.

• Main infrastructures adopted either collect centrally anonymized data or enable access to pseudonymized distributed data.

• Developing a common data model for storing all relevant information is a challenge.

• Trust of data providers in data sharing initiatives is essential.

• An online European Union meta-tool-repository is a necessity minimizing effort duplication for the various projects in the area.

**Supplementary Information:**

The online version contains supplementary material available at 10.1186/s41747-023-00336-x.

## Background

Artificial intelligence (AI) is transforming the field of medical imaging and has the potential to bring medicine from the era of disease categorization and population-based treatments to the era of personalized medicine, healthcare, and individual prevention. In particular, in the domain of cancer detection, treatment, and management, AI can solve several pressing and unmet clinical needs improving not only patient survival but also their quality of life. Worldwide interest in AI applications, imaging being one of the most prominent fields of application, is high and growing rapidly, fueled by the availability of large datasets (*big data*), substantial advances in computing power, and new deep-learning algorithms. Apart from developing new AI methods, there are many opportunities and challenges for the imaging community, including the development of a common nomenclator, better ways to store, curate, and share the now limited imaging datasets, and also standards for validating AI programs used across different imaging platforms and patient populations [1]. As such several initiatives have been established in the USA like the Cancer Imaging Archive (TCIA) [2], hosting a large archive of medical images of cancer accessible for public download and National Cancer Institute (NCI) Imaging Data Commons [3], offering a cloud-based data science platform for medical imaging.

In this direction, five EU projects (Primage [4], CHAIMELEON [5], ProCΑncer-I [6], INCISIVE [7], and EuCanImage [8]) are working together under the AI for health imaging (AI4HI) initiative, sharing experience and good-practices towards the development of big data infrastructures based on European, ethical and General Data Protection Regulation (GDPR) compliant, quality-controlled, cancer-related, medical imaging, and related patient’s data platforms, in which both large-scale data and AI algorithms will co-exist. The overarching vision of the platforms developed by these projects can be further specialized and made more concrete as follows:

- *Compliant with FAIR data principles:* the platforms should be built on FAIR-compliant and secure design and support data governance that enables sustainable cross-border connection of pan-cancer image data sources
- *Built for all involved stakeholders:* the designed systems should be primarily aimed for use by clinicians, researchers, AI modellers, and innovators. It is essential for such platforms to promote technologies and tools necessary for data analysis, in compliance with the relevant legal, ethical, quality and interoperability requirements and standards
- *Achieving interoperability and linkage of information:* the designed infrastructures should accommodate interoperability and linkage between fragmented data silos
- *Compliant with data quality standards:* support for actions aiming to create or extend cancer image data sources (including image annotation) and/or to adapt existing data (legacy data) in accordance with agreed data quality standards and legal requirements
- *Compliant with GDPR and legal requirements:* the platforms must be designed bearing in mind the data protection principles provided for by GDPR, while ensuring the integrity and confidentiality of the data. Functionalities of the platform need to take into consideration the Intellectual Property (IP) rights of the involved stakeholders, as well as the continuously evolving EU laws on data sovereignty, data governance and digital services
- *Offering high-performance computational and monitoring resources:* the platforms will offer high-end computing and simulation resources for data analysis, advanced feature-rich AI model development and evaluation environment, federated data analytics as well as a robust cyber-security layer.

In addition, such state-of-the-art platforms should, ideally, enable data linking and sharing among available EU research infrastructures and be able to interact and exchange data with other projects. Linking with such infrastructures requires common identity and access management, data, and model FAIRification. There are many challenges in trying to create such an infrastructure, and many obstacles should be overcome. This paper focuses on highlighting the various aspects involved in their design, present challenges, and potential solutions of the five projects positioning them based on the changes made for architecture, data models, GDPR considerations, and curation processes they adopt.

The remainder of this paper is structured as follows. First, we provide an overview of existing approaches to building such infrastructures for data storage, curation, and management for AI developments in cancer imaging, focusing on the data models used, on security aspects, and curation tools required from such infrastructures. Second, we present the common challenges of these projects. Finally, we present some conclusive remarks.

## Approaches of EU projects focusing on health imaging

In this section, we present the different approaches from the five EU projects of the AI4HI initiative. Overall, the projects use either centralized infrastructures where all data are stored in a central repository or federated infrastructures where data are stored at the hospitals and accessed on demand. Nevertheless, there are also approaches that adopt a hybrid architecture allowing both centralized and federated parts. On the other hand, for developing AI models on top of data, the data should be appropriately harmonized and homogenized. As such all projects adopt a common data model (CDM) (*e.g.*, OMOP-CDM) for the clinical data, including also standardized vocabularies (*e.g.*, SNOMED-CT, LOINC) for the data items to be stored there [9]. For imaging data, the DICOM standard is used. Additionally, all projects perform a series of data curation steps on the collected data in order to increase their quality and make them appropriate for training AI models on fop. Finally, all projects have to face the dilemma of fully anonymized data *versus* pseudonymized data for enabling GDPR-compliant data sharing.

### CHAIMELEON

CHAIMELEON aims to set up an EU-wide infrastructure, methodology, and tools to overcome the lack of availability of large quality controlled collected datasets and the heterogeneity of data and practices across institutions. Ultimately, it will enhance the reproducibility of radiomics features and achieve explainable AI for improved cancer management applications. These tools will be validated in the application context of four organs: lung, colorectal, breast, and prostate. CHAIMELEON adopts a hybrid architecture composed of both local data warehouses and a central repository with anonymized data. More details on the project architecture are provided in Additional file 1. The central repository will provide storage resources to share health images, related clinical data, and molecular data from pathology and liquid biopsy samples. Also, the repository will provide advanced computational cloud infrastructures to process these data as valuable resources for the AI community to develop and test practical tools (such as quantitative imaging biomarkers). Ιt includes various tools for curating the available data such as data completeness and consistency tools, image quality checking, image anonymization, annotation, segmentation, and harmonization. More details on the curation tools and approach for the project are provided in Additional file 1.

#### CHAIMELEON data models

Clinical data in CHAIMELEON are collected through electronic clinical report forms (eCRFs) and stored either in the local or in central database. OMOP was chosen as the Common Data Model for the local data warehouses and the structure of the eCRF. OMOP CDM is a commonly accepted international standard for working in observational studies, which facilitates interoperability between centers. OMOP CDM allows working with extensions, which gives greater adaptability when working with heterogeneous data. Within CHAIMELEON project the Oncology and Radiology extensions are used, actively contributing within the OHDSI community to the evolution of these data models and extensions. Harmonized datasets across all clinical sites are essential for AI training. In order to deal with the heterogeneity of practices across institutions, we proceeded in three steps:

- Radiologists defined the specific research question and inclusion/exclusion criteria for each use case and defined a list of variables to be collected for each type of cancer (lung, prostate, breast, colon, rectum)
- The eCRFs were designed for each cancer. As a first harmonization step, four main tabs were defined for each eCRF including one for patient general demographic information and three for the main disease-related time points: Diagnosis, treatment, and follow-up. Within each tab and each information entry, the structure was defined considering the requirements from the OMOP CDM v5.3 and the Oncology CDM proposal extension when applicable. Every concept proposed by the clinical experts was adapted to match the standard vocabularies and respect the specific domains required for each entry. Additional entries were added where necessary to ensure the availability of all mandatory fields. Tests of coherence were designed to help users verify inclusion/exclusion criteria and completeness
- Radiologists at each institution were asked to fill in test cases, to ensure they had access to all data and evaluate the time required to collect data for one patient.

#### CHAIMELEON: GDPR considerations and deidentification strategy

The project consortium decided to store anonymized data only on the central repository, facilitating the obtention of ethics committee approval at clinical sites. This is because even though the condition for this approval differs among institutions and regulations, providing anonymized rather than pseudonymized data to the project has proven to be a major key factor for ethical committee approval in most cases. The de-identification process is a two-step process:

- Step 1. Pseudonymization: DICOM images are pseudonymized using the following options with respect to the chapter “E. Attribute Confidentiality Profiles” of the DICOM Standard PS3 Part 15 [10]: (a) clean descriptors, (b) retain longitudinal full dates, and (c) retain patient characteristics. In this process, all direct identifiers are either removed or replaced by a randomly generated pseudonym. A table of correspondence is kept within the hospital and only accessible by an authorized user. Clinical data are associated with images using the same pseudonym. All dates, including exam dates and dates of birth, are kept at this stage. Keeping original dates at this stage proved to be necessary whenever discrepancies are found during the curation process, to allow back-and-forth discussions between people in charge of the data curation (pseudonymized data) and the ones in charge of the data collection (entitled to view identifying data).
- Step 2. Anonymization: Once the data curators have checked inclusion criteria appropriateness, data consistency, and completeness (at least 12 months of follow-up exams after treatment), a new patient identifier is generated (no table of correspondence kept), and all dates are shifted to keep longitudinal information. Only then, data is sent to the central repository.

### EuCanImage

EuCanImage is building a federated European cancer imaging platform that aims to improve AI capabilities in oncology. It is heavily based on major EU and international infrastructures, including Euro-BioImaging, ELIXIR, and its European Genome phenome Archive (EGA), BBMRI, and the Cancer Imaging Archive (TCIA). EuCanImage is a hybrid architecture composed of both local data warehouses and a central repository with anonymized data. More details on the project architecture are provided in Additional file 1. The infrastructure delivered will be populated with new data totalling over 25,000 single subjects focusing on unmet clinical needs in liver, rectal, colorectal, and breast cancer cases. Further, it includes various tools for curating the available data, and more specifically for image anonymization/pseudonymization, quality control and annotation, non-imaging data anonymization, and homogenization. More details on the curation tools and approach for the project are provided in Additional file 1.

#### EuCanImage data models

Within the project the following data models are used for the various data types:

- *Non-imaging data.* The ICGC (International Cancer Genome Consortium) ARGO (Accelerate Research in Genomic Oncology) dictionary was selected as a basis for the EuCanImage data model. It is a cancer-focused data model that describes the attributes and permissible values for all of the fields within the model.
- *Imaging data.* Based upon MIABIS-2.0 Core [11, 12] (Minimum Information About BIobank data Sharing) by BBMRI-ERIC [13], EuCanImage has adopted a similar model adapted for imaging metadata. This imaging model is a joint effort along with euCanSHare, a project for cardiac research from population cohorts in the EU and Canada. The metadata information is divided into three main levels, biobank, collection, and imaging datasets, where EuCanImage is displayed as a network of the biobank and/or collection. The metadata information is divided into three main levels, biobank, collection, and imaging datasets. The model focuses on image acquisition parameters, extracted imaging biomarkers, and post-processing tools and has been extended to add disease type at the collection level using the International Classification of Diseases, Version 11 (ICD-11) ontology for classification of disease diagnosis.
- *EuCanImage/EIBIR catalog.* The imaging catalog is built using the MOLGENIS [14] platform [15] (a modular web application for exploration of scientific data) and the user-search-interface is adapted from BBMRI-ERIC directory (molgenis-app-biobank-explorer) with the goal of future interoperability and sustainability. The user can search imaging metadata by network, country, the body part of interest, image modality, data types, collection types, and image access types. Each collection displays a table with the imaging datasets available and has the corresponding linking (URL and/or contact information) to their respective imaging and/or non-imaging data.

#### EuCanImage: GDPR considerations and deidentification strategy

Ensuring GDPR compliance principles is an ongoing effort in EuCanImage unfolding on three levels: (i) developing the policy framework for the platforms, (ii) creating data transfer and processing agreements governing data transactions, and (iii) translating legal and ethical requirements into technical ones and then implement these in the platform in a privacy by design fashion. More specifically, the following actions, tools, and measures will be implemented to ensure compliance with GDPR principles (art. 5):

- *Lawfulness, fairness*, and* transparency:* Data access procedures, implemented in the EuCanImage catalogue, will include the collection of justification for data use. Data access procedures will be enforced for imaging, omics, and phenotypic data, in accordance with GDPR principles via business rules. Through the catalog, each data provider will also guarantee the right to the information stated in arts 13 and 14 of the GDPR, which will be implemented as a binding term in data processing agreements.
- *Purpose limitation:* The overall purpose for data use in EuCanImage is scientific. Each data provider (data controller) will implement this requirement (as per art. 6.4 and 9.2 j) or ask for the data subject’s explicit consent for other purposes, where applicable. No secondary uses will be allowed without explicit consent.
- *Data minimization:* The selection of data strictly is based on the minimal data needed for the AI/clinical tasks.
- *Accuracy*,* storage limitation*,* integrity*, and* confidentiality:* Accuracy will be enforced starting with extensive data annotation and curation efforts. All patient-related personal data is removed within the clinic’s local network before upload to the platform. Patient health data as found in DICOM metadata is pseudonymized using SHA512/256. The pseudonymization key remains only visible to the source clinic. Associated consortium partners have no ability to reverse the pseudonymization. The pseudonymized data is guaranteed to be encrypted both in transit and at rest. Curators will check for, among other things, consistency and integrity in imaging and non-imaging data across sites. Revisions will focus on terms regarding the retention period, implementation of the right to be forgotten and other subjects’ rights, related policies, tools, and procedures.
- *Accountability* will be enforced primarily with the indication of data controllers, and type of controllership, at each step of the data flow as indicated in the data management plan and current architectural diagrams that are being reviewed.

### INCISIVE

INCISIVE aims to develop an interoperable pan-European federated repository of multimodal data sources, including imaging, biological, and other non-imaging clinical data is developed that enables the secure sharing of these data in compliance with ethical, legal and privacy demands, increasing accessibility to datasets, and enabling experimentation of AI-based solutions. INCISIVE adopts a hybrid federated architecture composed of both local data warehouses and a central repository with anonymized data. More details on the project architecture are provided in Additional file 1. Further, it includes various tools for curating the available data, and more specifically for image de-identification tools, quality control, and annotation tools. More details on the curation tools and approach for the project are provided in Additional file 1.

#### INCISIVE data models

**Fig. 1:**
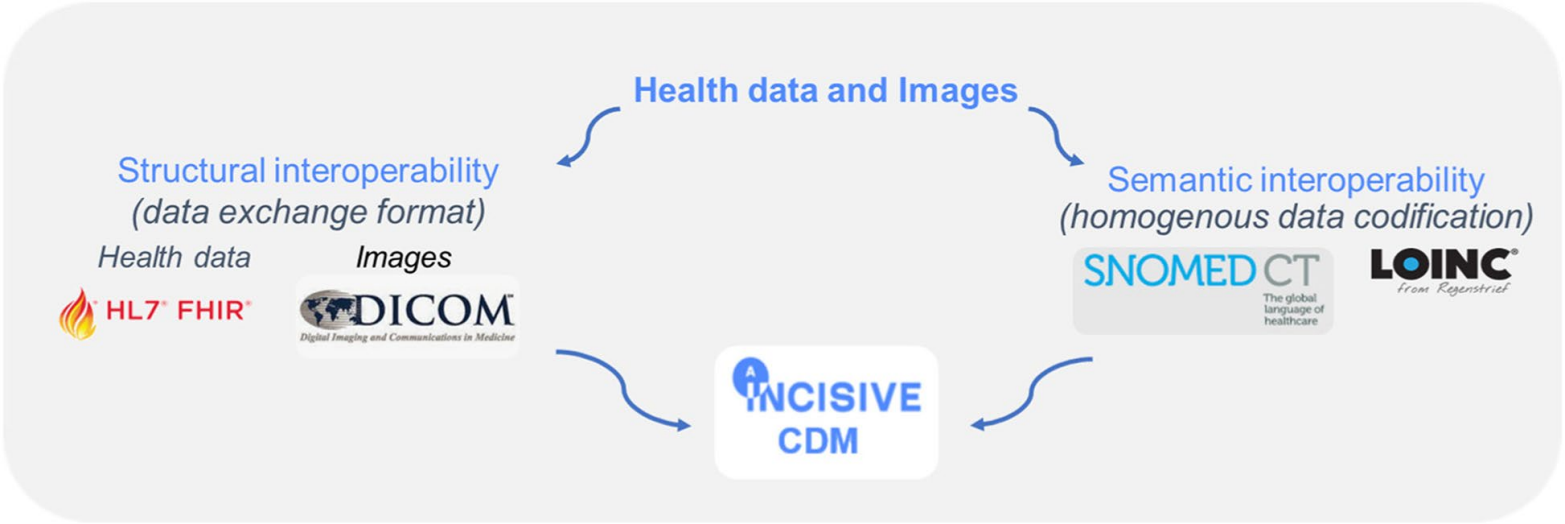
INCISIVE common data model backbone

**Fig. 2:**
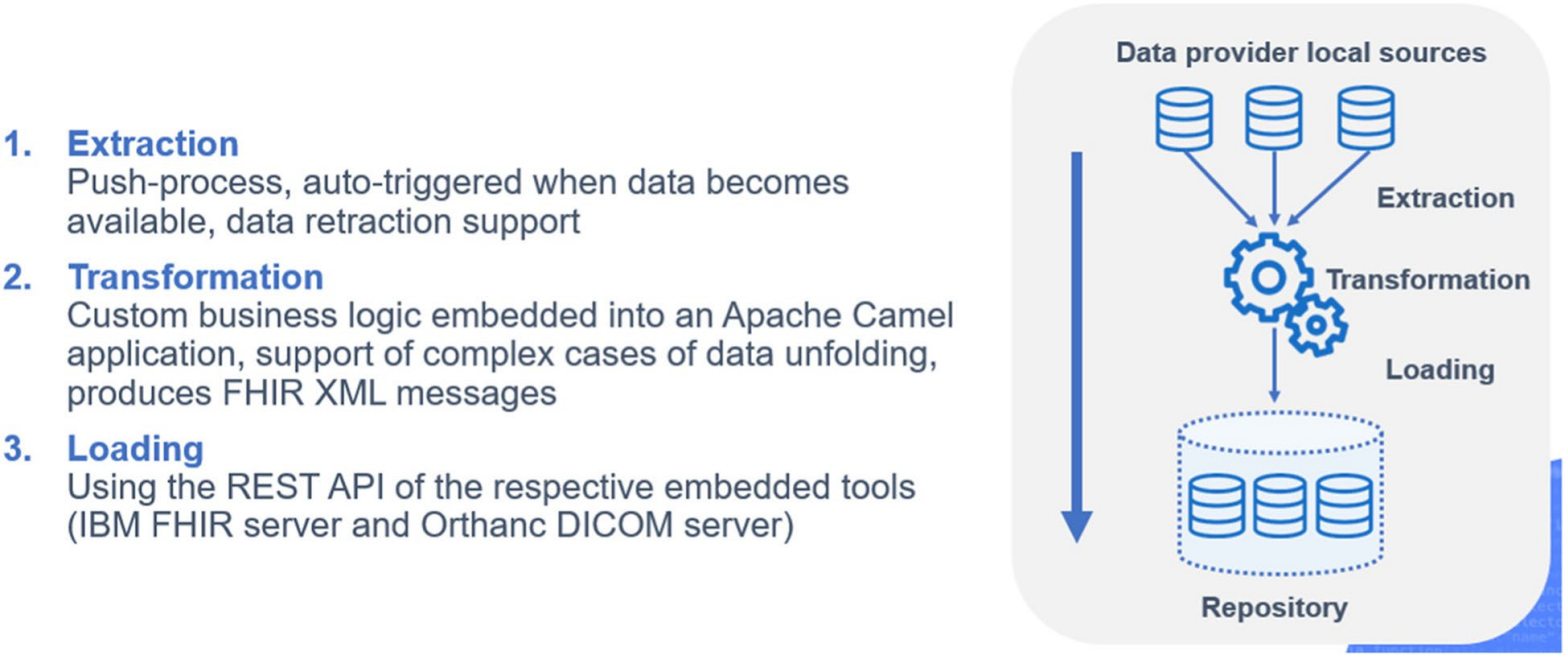
The Extract-Transform-Load (ETL) process in INCISIVE

The INCISIVE CDM builds on the FHIR and DICOM standards. FHIR is a standard for healthcare data exchange, published by Health Level Seven (HL7). The terminology adopted for semantic interoperability is based on the SNOMED ontology, the most comprehensive, multilingual clinical terminology in the world, and LOINC a universal standard for identifying medical laboratory observations (Fig. 1). A list of variables to be collected for each type of cancer addressed (colorectal, lung, prostate, breast) was built based on inclusion and exclusion criteria agreed upon with clinical experts. These variables have been mapped to SNOMED ontology for clinical data and LOINC when needed. Cancer data is collected into a Microsoft Excel template according to the aforementioned mapping and then is fetched from an Extract-Transform-Load (ETL) tool which reads them and generates FHIR messages (xml messages). Each FHIR message is sent to an underlying FHIR server, eventually loading all the data, which has been homogenized using the template, to the FHIR server. For images, INCISIVE follows a similar procedure with a second ETL tool that reads DICOM files placed in a directory and then sends them to an underlying PACS server. The PACS server integrates and enables the unified management of the DICOM images of all nodes in the INCISIVE infrastructure. Figure 2 illustrates the INCISIVE ETL process. In relation to the overall architecture, both ETL-FHIR and ETL-PACS components are provided as Docker containers.

#### INCISIVE: GDPR considerations and deidentification strategy

INCISIVE carefully considered the approach to de-identification of personal data in the project, taking into account not only the legal requirements, stemming from GDPR and relevant national laws, but also technical aspects of claiming that health data is irreversibly and “fully” anonymized and the limited utility of information altered to achieve anonymity for the AI training. The starting point of the considerations was related to use of retrospective data in the initial phase of the project.

INCISIVE recognized that GDPR does not mandate that only anonymous information is used for research. On the contrary, GDPR allows for personal data to be used in research, even if the data was originally collected for a purpose other than scientific research (“presumption of compatibility”), but such use must be subject to appropriate safeguards being put in place for the rights and freedoms of the data subjects. Further to GDPR, these safeguards should consist of implementing technical and organizational measures to ensure respect for the principle of data minimization, in particular pseudonymization of the data. The INCISIVE project also took into consideration that the national laws may impose additional conditions, in particular in relation to health data on the basis of the Article 9(4) GDPR. As a result, the project developed a set of safeguards for the protection of the rights and freedoms of the data subjects, including:

- Robust data pseudonymization process, by a dedicated de-identification tool and protocol (discussed in more detail below)
- Definition of processing roles and means of processing by clearly scoped joint controller agreement
- Data minimization by the methodology used to define the necessary retrospective data needed for the project
- Conducting Data Protection Impact Assessment (DPIA)
- Obtaining ethical approvals from local ethical committees for sharing of patient data
- Restricting access to data by multiple layers of security, including transfer of data over a secure server that providers access through a VPN connection; access to the data in the repository is provided only to a selected, verified group of researchers, on a need-to-know basis, and no data download is permitted.

The chosen method of pseudonymization allows data providers to make corrective changes to the data that will be submitted to the project repository and add new information that may not have been extracted (for instance, if some images or other data are found to be damaged -corrupted- when compressed). Having the ability to return to the specific data source reduces the possibility of errors and/or shortcomings and consequently increases the quality and accuracy of the algorithm to be developed and the quality of the project research outcomes. At later stages of the project, in particular in the context of submission of data to be shared beyond the repository via the federated/hybrid repository, the project is working on further going tools for de-identification of data, which will also allow data providers to submit data in an anonymous manner.

### ProCAncer-I

ProCAncer-I aims to create ProstateNET, a scalable, sustainable, quality-controlled, prostate-related, medical imaging platform, focusing on answering a number of important prostate cancer relevant clinical questions. These clinical questions have been the drivers for defining a set of nine use cases, which enable the creation of a unique dataset in terms of data quantity, quality, and diversity. ProstateNET promotes multi-center interoperability via a large multiparametric (mp) magnetic resonance imaging (MRI) data repository alongside tools for data analysis and sharing. Large-scale data (clinical and imaging) and artificial intelligence (AI) algorithms co-exist in a centralized architecture storing completely anonymized data. More details on the project architecture are provided in Additional file 1. Furthermore, ProstateNET includes various tools for curating the available information, and more specifically dedicated tools for image quality control, anonymization, motion-correction, co-registration, and annotation. More details on the curation tools and approach for the project are provided in Additional file 1.

#### ProCAncer-I data models

Within the ProCAncer-I project, the following data models are used for the various data types:

- *Non-imaging data.* Clinical experts defined all clinical, pathology, and follow-up data needed to be collected for every use case, which was reported in carefully designed eCRFs. Use cases refer to diagnostic and/or treatment-related challenges along the prostate cancer management continuum. All use cases have mandatory clinical information accompanying the imaging data (such as the PSA, biopsy, and/or prostatectomy confirmation of prostate cancer), and some other supplementary information depending on the use case. OMOP-CDM [16] is used to represent the clinical information which allows the standardization and harmonization of the information both on a structural level (data model) and terminological level (concept representation), enabling semantic interoperability as well as further enabling distributed network research and federated analytics. In addition, the oncology extension of the OMOP-CDM has been adopted, for representing the complex nature of cancer diagnosis, treatment, and cancer episodes at the levels of granularity needed to support oncology research. For all the basic clinical information, SNOMED-CT (and LOINC whenever that was not possible) has been adopted. For the oncology related information, SNOMED concepts augmented with ICD-O-3 were adopted for the base diagnosis, and NCIT, CAP, and NAACCR for the rest of the cancer diagnostic modifiers, all as part of the OMOP vocabularies (by using OMOP concept codes).
- *Imaging data.* ProCAncer-I adopts a radiology extension to the OMOP-CDM, in line with the OHDSI Medical Imaging Working Group efforts, which allows the standardization of essential DICOM metadata and enables the storage of data generated by subsequent annotation and curation processes. The radiology data model focuses on elevating and harmonizing the most important image acquisition parameters from the lower-level concepts of the DICOM instance metadata to a more abstract layer, for performing cohort definitions by using a combination of clinical and imaging parameters while maintaining provenance. The RSNA Radlex, a comprehensive set of radiology terms for use in radiology reporting, decision support, data mining, data registries, education and research, and SNOMED-CT have been used for representing the radiology procedure concepts (as OMOP concepts as well).
- *Metadata catalog.* Within the ProCAncer-I, the MOLGENIS [15] meta-data platform has been adopted to serve as the main metadata catalogue of the project. The user is able to search clinical and imaging metadata and retrieve cohorts by using a combination of all the different variables defined in every use case. All available information is connected with their corresponding imaging study, where the user can easily overview all the series (annotated, curated, or not).

#### ProCAncer-I: GDPR considerations and deidentification strategy

The ProCAncer-I project only intends to process personal data at the clinical level. Once data is shared with the consortium and used for the development of the project tool, then all data will have been anonymized and the GDPR no longer applies. However, until the anonymization has been completed, the GDPR applies to the processing of personal data including the pre-anonymization stage and in performing the anonymization. The ProCAncer-I anonymization strategy follows a double stage anonymization process. In the first stage, each partner uses the software tool of preference under the condition that it will successfully remove/modify the number of personal health information (PHI) tags as defined by the DICOM guidelines. At this stage, the issue of DICOM list heterogeneity still exists but the datasets are successfully stripped out by any PHI containing tags. The second stage of anonymization addresses the problem of DICOM tag list heterogeneity (different MRI scanner vendors and software versions), and the existence of the private tag list. This process is performed by the well-known RSNA utility [17] embedded in the ProCAncer-I.

### PRIMAGE

The PRIMAGE project [4] aims to develop a cloud-based platform to support decision making in two main pediatric cancers, neuroblastoma (NB), and Diffuse Intrinsic Pontine Glioma (DIPG). It adopts a centralized architecture storing completely anonymized data. More details on the project architecture are provided in Additional file 1. Further, it includes various tools for curating the available data, and more specifically for image labeling, quality checking, annotation, denoising, motion correction, and registration. More details on the curation tools and approach for the project are provided in Additional file 1.

#### PRIMAGE data models

Within the PRIMAGE project, the MIABIS data model is used. However, as it is specifically developed for traditional biobanks, it is not suitable to fully describe imaging biobanks, which contain a much different type of data (*i.e.*, medical images and associated metadata describing the images, their analysis, and the patient’s medical history). Within the PRIMAGE project, MIABIS was extended specifically for imaging biobanks, in order to better represent this type of data while still maintaining a connection with the standard defined by MIABIS. The first version of the model suggests replacing the MIABIS entity “sample collection” with a more general “collection,” which is then linked on one side to the MIABIS entities, and on the other to newly defined entities specific to images [18]. PRIMAGE is currently focusing on the completion of this model, in particular by adding the missing entities and attributes related to the patient clinical variables, which were not yet defined in the first version, and by mapping these clinical variables to the OMOP CDM. Finally, the DICOM-MIABIS model is being used to store all the non-clinical information, this includes image preprocessing and annotation parameters, radiomics features, or DICOM metadata; however, for clinical data, the OMOP CDM is being used. The PRIMAGE database is natively built under a noSQL MongoDB database where all the information is stored in JSON format. Therefore, to map all the information into the proposed data models an ETL process is being developed.

#### PRIMAGE: GDPR considerations and deidentification strategy

PRIMAGE deals with real-world data from NB and DIPG patients that have already been diagnosed and treated in different collaborating hospitals, registries, and trials to develop the in silico tumor behavior prediction models. In the project, access is granted to several registries and clinical trial databases for secondary use of available clinical data. The databases included are the SIOPEN-r-net (International Society of Pediatric Oncology European Neuroblastoma Research Network) and the GPOH clinical trials database (German Society of Pediatric Oncology and Hematology). The secondary use of clinical trial data must necessarily be done in agreement with the Ethics Committee and highly respecting data protection rules. For the external validation of the developed algorithms, other hospitals will collaborate with PRIMAGE sharing their data. The ethics committee approval of these external collaborators is mandatory.

Data from the SIOPEN-r-net database was already pseudonymized using EUPID (European Unified Patient Identity) [19]. To ease the linkage between external databases and the PRIMAGE database EUPID was also included as the pseudonymization tool in the PRIMAGE project. As detailed in the previous section, data comes from different sources following different data upload pipelines. Regardless of the source of the data, when a new patient is incorporated in the PRIMAGE database, a new and unique pseudonym is given for its pseudonymization. When uploading an associated imaging study, this pseudonym is used to substitute personal data in the DICOM files such as the patient name or the patient ID and all the DICOM tags with sensitive information as stated in the DICOM standards PS3.15 [20] are removed or emptied from the uploaded files.

## Common challenges

The key dimensions of the infrastructures, data models, deidentification, and curation process developed by the AI4HI projects are shown in Table 1. While there are some commonalities to all projects, different approaches were adopted for all the dimensions of the projects. In the sequel, we discuss some key commonalities and differences:

**Table 1:** The key dimensions of the infrastructure developed for each one of the five AI4HI projects

	CHAIMELEON	EuCanImage	INCISIVE	ProCAncer-I	PRIMAGE
Cancer types	Lung Colorectal Breast Prostate	Colorectal Liver Breast	Lung Colorectal Breast Prostate	Prostate	Neuroblastoma Diffuse intrinsic pontine glioma
Architecture	Hybrid	Accommodating both decentralized and centralized storage	Hybrid (federated and centralized storage)	Centralized	Centralized
Data models and types of data	DICOM-MIABIS OMOP CDM (terminology IDs mainly) Structure of the eCRF	DICOM-MIABIS FHIR (and terminologies supported by FHIR + extensions)	FHIR SNOMED-CT LOINC DICOM	DICOM-RT OMOP CDM with extensions	DICOM-MIABIS OMOP CDM
Deidentification process	Pseudonymized initially for curation and then fully anonymized data at the central repository	Pseudonymized data	Pseudonymized data	Fully anonymized data	Pseudonymized data
Curation tools	Data completeness and consistency tools, image quality checking, Image anonymization, annotation, segmentation and harmonization	Image anonymization/pseudonymization, quality control and annotation, non-imaging data anonymization and homogenization	Image de-identification tools, quality control, and annotation tools	Image quality control, anonymization, motion-correction, co-registration & annotation.	Image labelling, quality checking, annotation, denoising, motion correction, registration
Number of potential subjects	13,000 full cases (images + clinical data), 34,000 image only cases	25,000	8,850	17,115	1,500
Number and type of clinical sites	8 university hospitals	6 hospitals (including 4 university hospitals)	9 data providers (5 universities associated with 5 external hospitals, 2 hospitals, 1 association, 1 company)	13 data providers	Clinical trials: HR-NBL1, LINES and Society of Paediatric Oncology and Haematology. 16 external hospitals.
Location of the central repository, when applicable	Universitat Politècnica de València, Spain	European Institute for Biomedical Imaging Research/EIBIR	MAGGIOLI, Greece	FORTH-ICS, Greece	Microsoft Azure, Dublin, Ireland & Universitat Politècnica de València, Spain
Level of completion (maturity) of the project	+8,500 patients screened	Data deposition & annotation started end of 2022.	Retrospective data collection and annotation finished, prospective data collection finishes end 2023, storage platform almost ready storing all available data so far. The project finishes in March 2024	Retrospective data collection almost finished. Platform almost ready. AI model development started.	Platform ready. Closing data incorporation (cases for external validation) and AI models tunning and testing. The project finishes in May 2023
Involvement of industry	SMEs: Medexprim, Quibim, Bahia Large corporation: General Electric	SMEs: Collective Minds Radiology, Lynkeus, Radiomics. Large corporation: Siemens	SMEs: European Dynamics, Telesto, Squaredev, ADAPTIT, VISARIS, Timelex, White Research Large corporations: MAGGIOLI, Medtronic	SMEs: B3D, Advantis, Quibim Large corporation: Quiron	SMEs: Quibim, Medexprim, Chemotargets.

*AI* Artificial intelligence, *DICOM* Digital imaging and communications in medicine, *eCRF* Electronic clinical report form, *FHIR* Fast Healthcare Interoperability Resources, *HR-NBL1* High-risk neuroblastoma 1 trial, *IDs* Identifiers, *LINES* Low and intermediate risk neuroblastoma, *LOINC* Logical observation identifiers names and codes, *MIABIS* Minimum Information about Biobank Data Sharing, *SMEs* Small-medium enterprises, *SNOMED-CT* systematized nomenclature of medicine clinical terms

### Architecture

Two projects (ProCAncer-I and PRIMAGE) opted for a centralized architecture. CHAIMELEON and EuCanImage’s architecture is hybrid, and INCISIVE is mainly federated, although now moving towards a hybrid approach. Centralizing data provides efficiency in data exploration and processing, while a federated approach provides data holders with additional control and reassurance over data privacy and data sovereignty. Taking into account the advantages and disadvantages of the different architectures, a hybrid approach seems to be the best one, combining the advantages of both centralized and federated architectures. In a hybrid approach, data is centralized only when required after data sharing has been authorized by the data providers. This approach allows combining the ability to have very large data collections locally stored in data warehouses at sites and the ability to make use of real-time cloud high-performance resources for specific uses and specific datasets. Whichever approach, a common data model is necessary.

### Data models and standards

While DICOM is a universal standard for medical imaging and was adopted by all projects, clinical data suffer from greater variability. Several ontologies or data models coexist and projects combine the OMOP-CDM, SNOMED-CT, HL7 FHIR, or ICGC-ARGO. Multiple terminologies are used to further homogenize various fields. It is also worth mentioning that although the objectives of each project were close, each project defined the clinical variables they wanted to collect and each project developed its eCRF. Nevertheless, all projects work on existing current terminologies and standards to fill the gaps for medical imaging. Metadata used to describe a dataset also varies from project to project, although all projects refer to the MIABIS recommendations and the MIABIS-DICOM proposition. EuCanImage and ProCancer-I both use the MOLGENIS platform to create metadata for the datasets. This variability in data models shows the need to have clear guidelines and for all projects to work together to extend existing terminologies and standards and to fill gaps. A common data model and common terminologies are required to respect true FAIR principles and promote interoperability.

### Cloud agnostic *versus* cloud dependent solutions

For deploying and ensuring the sustainability of the generated infrastructures they should be based on cloud infrastructure services with workload portability, providing the capability to create a service package and then be able to provide that package in different cloud service providers without substantial modifications. The management services of cloud infrastructure shall, where possible, be based on open standards adopted by major cloud providers to allow the required interoperability and independence from vendors. However, this is usually a step difficult to be implemented in practice as each cloud provider has its own offerings in order to engage and commit the clients to a specific infrastructure. However, this is something that should be paid proper attention to for the projects that implement such infrastructures in order to keep their options open for the future.

### GDPR considerations

There is no “one-size-fits-all” solution to ensuring the appropriate legal basis for the processing of health data for scientific research. All projects have appointed a Data Protection Officer; have determined the roles of the different stakeholders; have formalized arrangements such as joint controller agreements data processing agreements and data transfer agreements; have run a data protection impact assessment (DPIA); and have ensured that strict privacy guarantees are in place. Some aspects may differ however from project to project and some GDPR aspects may go against some objectives of the projects. Below are some specifics that have led to discussions within the projects, calling for the need to have clear guidelines.

### Consent *versus* patient information

While the consent of the patients may be praised for having the benefit of transparency for the data subjects, in real-life scenarios related to the use of retrospective data obtaining such consent may not be possible. What is commonly accepted for all projects is the legitimate use of the data and waiver to obtain formal patient consent. The means to deliver patient information, however, may differ from country to country, or from institution to institution. In some cases, individual information is delivered for the specific project, but in most cases, institutions deliver patients with general information on the possible use of their data for research and modalities for the patient to exercise their access or opposition rights. They maintain a list of research projects that patients can consult. The authorization to share data is usually delivered by the institution’s Ethical Committee, which assesses the legitimacy of the research project and the data to be accessed, and the institution’s Data Protection Officer, who evaluates technical and organizational measures to ensure data privacy. Each of the projects has delegated to the clinical partners the responsibility to obtain the relevant authorizations from their institution, based on descriptions of the project, the requested data, and the data processing.

### Accountability

While data protection impact assessments (DPIA) allow to achieve baseline compliance and drive a discussion on the discovered risks to privacy and means to mitigate them, observing the principle of accountability in large consortia is difficult, as there are many different stakeholders which take responsibility for various aspects of processing. The new legal frameworks such as the Data Governance Act and the European Health Data Space will be of great help to set up data governance structures, based on an in-depth mapping of the roles of the stakeholders (data providers, data users, and infrastructure administrators) and careful balancing of their interests and liabilities.

### Data minimization

The balance between data minimization principles and the objectives of most projects to have as much data as possible is difficult to achieve. There is no standard or consensus on what amount and variability of data is required and sufficient in AI projects to have a high enough level of confidence in the reliability of models and the absence of biases. As the projects move forward and assess the quality and quantity of data, there will be an opportunity for the five projects to work together and propose guidelines on what is the right level of data collection to achieve both the objectives of the research projects and respect data minimization principles.

### Deidentification

While all projects have adopted strict de-identification measures, based on the confidentiality profile defined by the DICOM Standard, and have taken both technical and organizational measures so that data users cannot reidentify patients, there are significant differences in approaches. Some projects have opted for pseudonymization, while others perform anonymization. In pseudonymization, the data provider keeps the key to the patients’ original identity. This has several advantages:

- It facilitates the curation process where authorized users can access the EHR data to verify the data completeness and address any doubts when dealing with outliers or incoherent data
- Additional time points can be added to the data and are linked with previously collected information
- Patient withdrawals can be handled
- Patients can be reached by caregivers in case of incidental findings in a research project.

In anonymization, deidentification is irreversible. No table of correspondence is kept, even at the hospital level, so it is no longer possible to identify a person. Anonymous data is no longer considered personal data. Therefore, anonymous data falls outside of the scope of GDPR. For this reason, anonymization may be preferred by some institutions, as otherwise, it would be difficult for them to assume any responsibility over personal data that are stored in an environment that is not managed by them. Anonymization, however, requires specific precautions:

- As it is not possible to add timepoints, only full cases with longitudinal information and evaluation during full follow-up period can be sent
- Specific measures need to be taken to ensure no duplicates of patients are sent
- The European Data Protection Board (EDPB) has warned that anonymization is difficult to achieve and maintain over time “taking into consideration the available technology at the time of the processing and technological developments”.^1^ Additional safeguards should be taken to ensure reidentification would be impossible to achieve, such as ensuring that downloading data is not possible and that any further processing of the data is monitored and justified.

Standardized guidelines would avoid individual projects having to make a choice and would reassure data holders.

### Curation process

Finally, multiple curation tools are available from all projects, namely tools for checking the image quality, anonymization, annotation, segmentation, co-registration, motion correction, and denoising. While the general curation tasks are the same in all projects and while the need for high-quality, annotated, curated data is crucial for various research and AI experimentation purposes, there is a great variability in the methods chosen. As such, there is a high need to streamline tools and methods for data curation for the data to become useful for various research and AI experimentation purposes.

## Conclusions and perspectives

Developing high-quality AI models for health imaging requires access to large amounts of curated, annotated, and harmonized imaging data along with their metadata and associated clinical labeling information. However, these datasets might have been produced by different vendors and protocols and use different terminologies and data models to be represented. As such, appropriate infrastructures are required for enabling access to the data and raising the trust of data providers in data sharing initiatives. Various approaches have been implemented already by the projects participating in the AI4HI initiative, ranging from centrally collecting anonymized data to federated learning performed at each clinical center. Independent of the approach, a harmonization methodology is also required to ensure homogeneous access to heterogeneous data. Further, despite the plethora of models, typically the specific requirements set by each project necessitate the use of specific models and terminologies in order to appropriately describe the available data making the result data from each project not directly homogenized with data from the other projects. And even that is usually not enough, as extensions are also often required that are project-specific. On the other hand, based on the specific data collected by each project there are also corresponding curation tools. The requirements for data curation, however, lead to similar tools, and efforts are duplicated across the different projects. As such we envision an online EU meta-tool-repository where the tools will be available to be used directly by the various projects working on the area, without requiring effort duplication.

To further harmonize practices, contribute to standards, ensure interoperability and in-fine, raise trust in data sharing, and advance AI development in cancer imaging, the five projects of the AI4HI initiative have gathered in a new project: EuCAIM (European Federation for Cancer Images) funded under the Digital Europe Programme. This very ambitious project, composed of 79 partners, will demonstrate technologies, operational procedures, and legal frameworks which are reproducible, efficient, and cost-effective for enabling a cross-border reuse of health data in research and innovation projects.

## Supplementary Information

**Additional file 1:** Architectures and curation process for the five projects. **Fig. S3.** The overall CHAIMELEON architecture. **Fig. S4.** General overview of the CHAIMELEON Central Repository Architecture. EuCanImage. **Fig. S5.** Main components of the EuCanImage’s data management platform. **Fig. S6.** Schematic structure of data flow when a central storage (a) and distributed storage (b) model is used. INCISIVE. **Fig. S7.** INCISIVE preliminary architecture. **Fig. S8.** Illustration of DICOM metadata and burned-in annotations de-identification. **Fig. S9.** Data flow pipeline. ProCAncer-I. **Fig. S10.** The main subsystems of the ProCAncer-I platform. **Fig. S11.** The main subsystems of the ProCAncer-I platform. **Fig. S12.** Data acquisition flow. PRIMAGE. **Fig. S13. **PRIMAGE web platform architecture and connection with HPC cloud infrastructure. **Fig. S14.** Data flow for imaging data upload to the PRIMAGE central repository [21–30].

## Data Availability

Not applicable.

## Abbreviations

AI: Artificial intelligence
AI4HI: AI for health imaging
DICOM: Digital Imaging and Communications in Medicine
DIPG: Diffuse Intrinsic Pontine Glioma
DPIA: Data Protection Impact Assessment
eCRF: Electronic Clinical Report Form
ETL: Extract-transform-load
EU: European Union
FAIR: Findable, Available, Interoperable, Reusable
FHIR: Fast Healthcare Interoperability Resources
GDPR: Regulation (EU) 2016/679 of the European Parliament and of the Council of 27 April 2016 on the protection of natural persons with regard to the processing of personal data and on the free movement of such data, and repealing Directive 95/46/EC (General Data Protection Regulation)
HL7: Health Level Seven
LOINC: Logical Observation Identifiers Names and Codes
MIABIS: Minimum Information about Biobank Data Sharing
MRI: Magnetic resonance imaging
NB: Neuroblastoma
OHDSI: Observational Health Data Sciences and Informatics
OMOP: Observational Medical Outcomes Partnership
PHI: Protected/Personal Health Information (any information in a medical record that can be used to identify an individual)
SNOMED-CT: Systematized Nomenclature of Medicine Clinical Terms
TCIA: The Cancer Imaging Archive

## Footnotes

1
https://edpb.europa.eu/sites/default/files/files/file1/edpb_replyec_questionnaireresearch_final.pdf
