# Supplementary material for: Data infrastructures for AI in medical imaging: a report on the experiences of five EU projects
Source: Eur Radiol Exp. 2023 May 8;7:20. doi: 10.1186/s41747-023-00336-x (PMC10164664; doi:10.1186/s41747-023-00336-x)
Supplement: Supplementary file 1 — Additional file 1: Architectures and curation process for the five projects. Fig. S3. The overall CHAIMELEON architecture. Fig. S4. General overview of the CHAIMELEON Central Repository Architecture. EuCanImage. Fig. S5. Main components of the EuCanImage’s data management platform. Fig. S6. Schematic structure of data flow when a central storage (a) and distributed storage (b) model is used. INCISIVE. Fig. S7. INCISIVE preliminary architecture. Fig. S8. Illustration of DICOM metadata and burned-in annotations de-identification. Fig. S9. Data flow pipeline. ProCAncer-I. Fig. S10. The main subsystems of the ProCAncer-I platform. Fig. S11. The main subsystems of the ProCAncer-I platform. Fig. S12. Data acquisition flow. PRIMAGE. Fig. S13. PRIMAGE web platform architecture and connection with HPC cloud infrastructure. Fig. S14. Data flow for imaging data upload to the PRIMAGE central repository [21–30]. [file 41747_2023_336_MOESM1_ESM.pdf]

ELECTRONIC SUPPLEMENTARY MATERIAL

Data infrastructures for AI in medical imaging: a report on the experiences of five EU projects

Appendix 1 - CHAIMELEON

1.1 Architecture

The CHAIMELEON architecture is a hybrid architecture (Figure 3), and is composed of:

- Local data warehouses and tools deployed within the hospitals to streamline the process of data collection, curation, and anonymization (*Medexprim Suite™* provided by Medexprim).
- The central repository to manage and annotate anonymized data, train AI models, and experiment processing pipelines (hosted and provided by Universitat Politècnica de València, with the front end based on Quibim Precision®, provided by Quibim).
- A national intermediation platform for French sites provided by Medexprim, receiving pseudonymized data from French hospitals and used by data managers from CERF (Collège des Enseignants de Radiologie Française) to curate data before it is sent to the central repository.

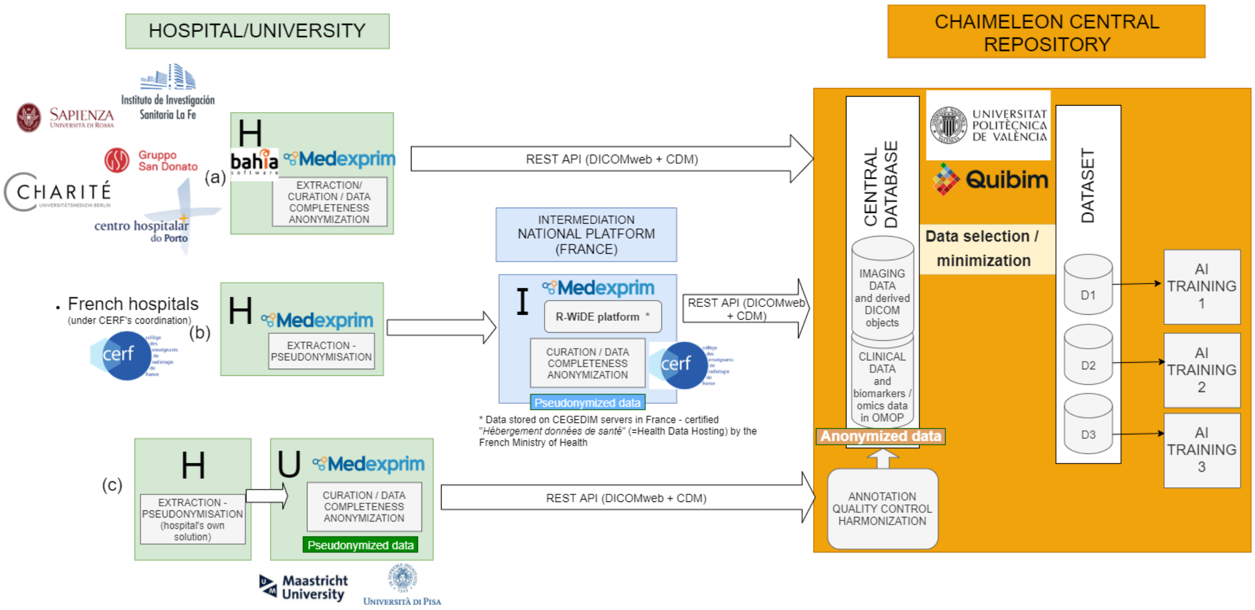

Figure 3. The overall CHAIMELEON architecture.

The *Central* Repository is composed of four main blocs (Figure 4):

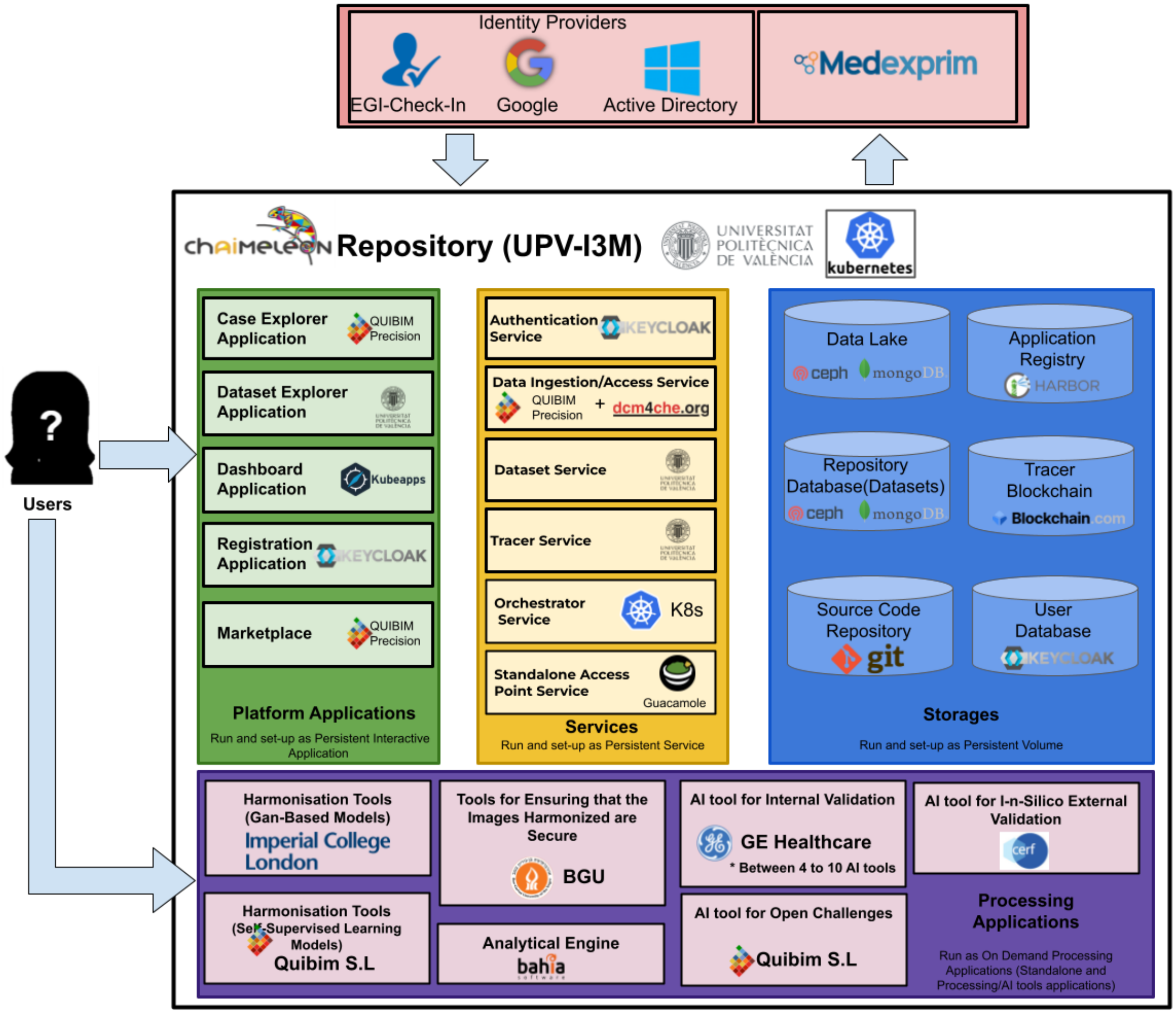

Figure 4. General overview of the CHAIMELEON Central Repository Architecture.

- There are two types of Processing applications: The Standalone Applications and Processing and AI Tools. Standalone Applications are deployed on-demand to train AI models using the datasets selected through the Dataset Explorer Application. The Processing and AI Tools are applications integrated into the Marketplace and the Case Explorer based on the trained model, such as Harmonisation tools, analytical engines of data, etc.

- Platform Applications are the services that provide the functionalities (user actions) offered by the Central Repository through user-friendly interfaces. Users only can interact with the Central Repository through these applications: Case Explorer and Marketplace (Quibim Precision®), a Dataset Explorer application (UPV), a Dashboard Applications (Kubeapps) and a Registration application (Keycloak).
- Services are the principal components of the Central Repository. Some of them will be internal services for the administration of the infrastructure, such as the Authentication Service (Keycloak) or the Container Orchestrator (Kubernetes) for deploying Processing Applications. Others such as the Access/Ingestion service or the Standalone Access Point Service (Guacamole) permit the interaction with other Repository applications offering specific functionalities such as the data Ingestion in bulk mode. The data ingestion/access service allows external applications like Medexprim's instances to feed the platform using the DICOM Web protocol for images (dcm4che server) and a REST API for clinical data in JSON format, through Quibim Precision® back-end. The Dataset Service (UPV) and the Tracer service (UPV) manage the datasets lifecycle and track the user actions performed with datasets at the Central repository.
- Storages provide data persistence (including medical data, application binaries and specifications, traceability logs, users' profiles) in the CHAIMELEON Central Repository. Storages are uniquely managed by Services or Applications and cannot be accessed directly by the end-users.

1.2 Curation Process

The curation process is a several-step endeavour, with some steps done on pseudonymized data on Medexprim's *Intermedia*™ module, within the clinical sites for the most part, and other steps done on the central repository on anonymized data using tools provided by Quibim Precision®. More specifically, the following tasks are done by data curators on *Intermedia*™ on pseudonymized data before data gets anonymized and sent to the central CHAIMELEON repository:

- **Clinical data completeness and consistency:** While inclusion criteria and some data consistency is done at time of data collection through consistency rules programmed within the eCRF, more control is done on the data after collection. In particular, graphs show data distribution and help detect outliers or missing data. In case of a suspected error

or missing data, the data curator can raise a "query" for resolution by the data collector. Because this is done on pseudonymized data, the data collector can check and correct the data, as she/he has access to the pseudonymization table and the identifying data in the EHR.

- **Detecting patient identifying information in image pixel data:** the pseudonymization process within *Radiomics Enabler*® processes the image DICOM headers (metadata) and, by default, filters out any DICOM object that is prone to identify data in pixels; that is: secondary captures, images from specific modalities: and DICOM SOP classes [21]. If some of these DICOM objects are required for the project, it is possible to let them through and configure a "pixel anonymizer" that will apply a cache on the pixels to blank patient identification. Despite all the care in applying careful deidentifying algorithms, it may happen that some images with remaining patient information in pixel data go through. We developed a solution that can provide a view through the whole series data, so that an operator can detect at a single glance if an image with text is present within the series. The curator can then delete the image or the whole series if it is not required for the project or raise a query to the attention of the data collector for correction.
- **Checking image quality**: Image quality is checked using the same method as described previously. If an exam is not of sufficient quality, the data curator can raise an issue so that the data collector can see if there exists an alternative exam. If not, the patient can be excluded.

The tasks that do not require access to the original data are then done on the CHAIMELEON central repository on anonymized data using the tools provided within Quibim Precision® together with other tools developed by the different partners in the context of the project and integrated into the same platform. These tools include:

- **Image annotation and segmentation:** Quibim Precision® platform includes an image annotation environment for the delineation of regions of interest. A DICOM study can be loaded and explored in this environment and, using some manual and semi-automatic tools, different regions of interest can be delineated. In addition, the platform allows for the tagging of these imaging studies using MESH, Radlex or custom tags.
- **Image harmonization:** Access to large volumes of health imaging datasets will imply the use of data acquired at different centres with different scanners and acquisition protocols. Due to this reason, the quantitative imaging features extracted from images acquired at one centre may not be reproducible from images acquired in another centre,

due to a lack of consistency of source medical images generated from different equipment vendors, models and releases as well as to the lack of an appropriate framework in terms of image acquisition/reconstruction, pre-processing and workflow steps. To overcome these drawbacks, different harmonization approaches based on AI to generate synthetic images adjusted to a common harmonization framework have been proposed. Specifically, two different approaches are being explored. On one hand, Generative Adversarial Networks (GAN) [22, 23] to transform images from different domains to one specific domain (harmonized) are being used. On the other hand, self-supervised learning approaches are being explored, these are mainly investigating the use of the images frequency domain to reconstruct images with common intensity values.

Appendix 2 - EuCanImage

2.1 Architecture

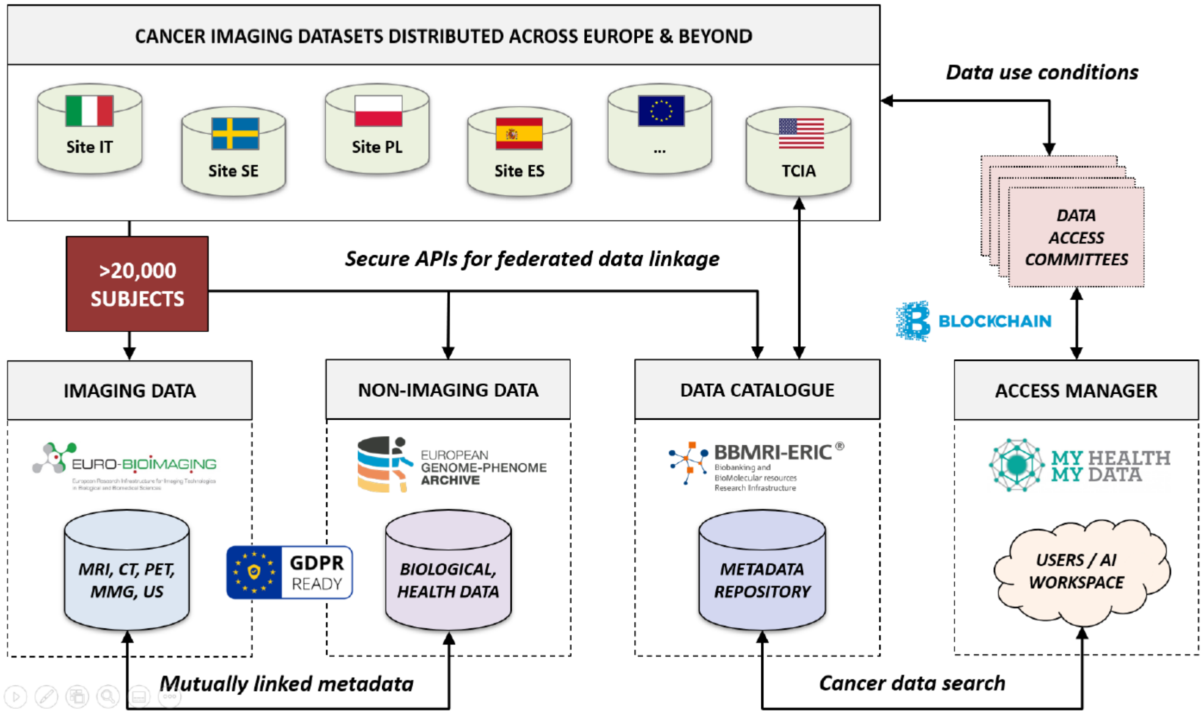

Figure 5. Main components of the EuCanImage's data management platform.

The EuCanImage includes the following components as shown in Figure 5: a) Data Storage components (Euro-BioImaging and EGA); b) Data Browser module (Cohort Browser) that will allow users to locate data of their interest; c) Data Access subportal to request access to selected data; d) Access Manager that will handle actual access to the data; e) Workspace that will allow users to submit data they have been granted access to for quality control, harmonization or analysis to visualize and download results; f)Data Analysis module. In the sequel, we provide more details on the core components.

Data storage. Within EuCanImage, two approaches for data storage will be supported, a centralized and a distributed one[1]. In the centralized approach (Figure 6a), data will be hosted at two specialized repositories: EGA (non-imaging data) and Euro-BioImaging (imaging data). The centralized approach facilitates the processes of annotations, curation and de-identification. In the distributed approach (Figure 6b), the data will be hosted locally, without leaving the hospitals. This means that all data hosting and curation must be done locally and the development and application of AI models has to be done in a distributed / federated manner, also called federated learning. The choice between central or distributed storage mainly depends on the regulations, needs, and availability of local (IT) support of the data providers. The designed infrastructure facilitates not only interoperability between the two approaches, but also the combination of central and distributed datasets in the AI model development.

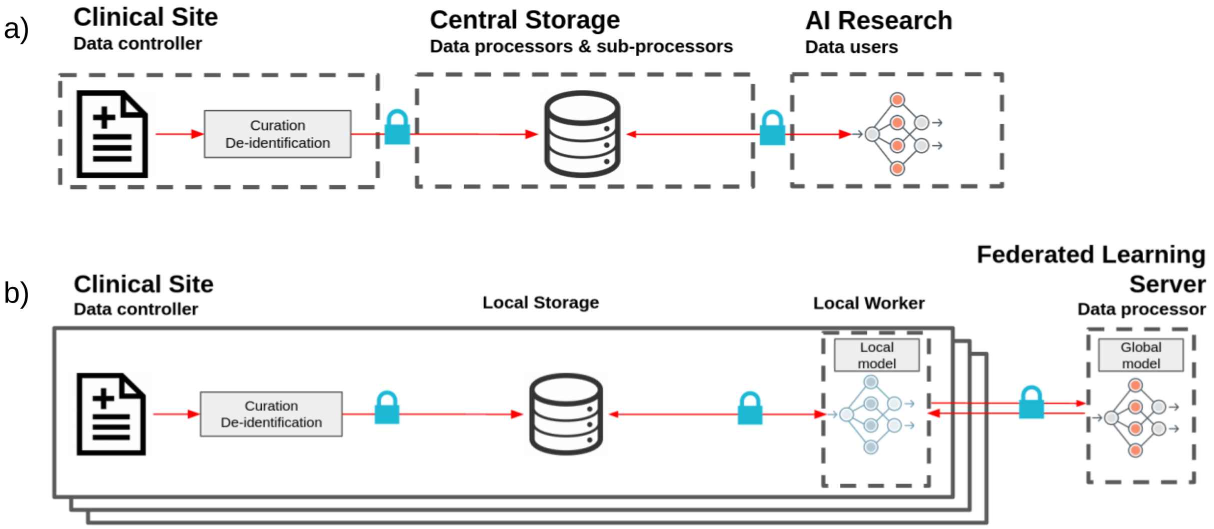

Figure 6. Schematic structure of data flow when a central storage (a) and distributed storage (b) model is used.

[1] It is important to remember that this applies to data storage, not management. The data providers will indeed, in any case, remain data controllers.

Data analysis. Depending on the data location and access requirements, as well as the processing computational needs, this module offers several complementary analysis setups. The AI research environment (AI-VRE) is part of the Data Analysis module. AI-VRE will present the researchers with a computational environment to process data using a collection of cancer radiomics tools and a complete machine learning toolbox for integrated predictive modelling. The framework, fully virtualized, might be installed centrally or in a particular data centre. When installed centrally, the researcher's datasets that are made accessible for analysis, will be either uploaded through the web (HTTPS) from a local device, or imported from the primary data storage (i.e., XNAT, EGA), always bound to the data access requirements applicable on each dataset. Hence, when interacting with the repository controlling the data, the AI-VRE is acting on behalf of the researcher, who has agreed on delegating authentication to the AI-VRE system. When installed on individual data centres, data volumes accessible on the local network become also eligible for analysis, so that protected datasets are kept within premises. Data processing will occur on the in-premises cloud resources on top of which AI-VRE is installed, unless remote executions are enabled to distribute workload among federated clouds.

2.2 Curation Process

In the EuCanImage project both imaging and non-imaging data are collected per subject. This combination results in a broader spectrum of data which in turn is expected to bring forth better performing AI models. Each type of data has its own curation process. In the sequel we provide details on this curation:

- **Imaging data curation:** When curating images within the EuCanImage the data will undergo the following steps: pseudonymization, quality control and annotation. Within the project there are two ways to achieve de-identification; through the use of RSNA Clinical Trial Processor (CTP) [24] in combination with POSDA [25, 26] from TCIA, as well as CM-Proxy from Collective Minds Radiology (CMRAD) [27]. For the former solution, CTP will perform the pseudonymization and an initial anonymization step, after which POSDA is used to manually check and correct the information in the DICOM headers and the images themselves in an easy manner. After pseudonymization and anonymization with CTP and POSDA, the latter will also be used for the quality control step. POSDA offers various checks to, for example, assure the validity of the DICOM file and remove any duplicates within the dataset. Within POSDA, tools are available which

will be used to perform a visual check to assure the quality of the images. If CM-Proxy is applied for this step, the images will afterwards be sent to the CMRAD platform where a visual check of the images can take place. The use of the CMRAD platform and the POSDA software are not mutually exclusive. After using CM-Proxy and the CMRAD platform, the images can be retrieved and processed by POSDA. After POSDA is used for the curation of the data, the images can be sent to the CMRAD platform for annotation. For using the images for AI models, annotation and segmentation are performed using the CMRAD platform. Within the project, a combination of full segmentations, segmentations with bounding boxes as well as labels on the subject and lesion levels are produced, depending on the use case. After annotation, the image is retrieved from the CMRAD platform and stored on the EuroBioImaging XNAT, when using the centralized data storage, or locally, for use in federated learning.

- **Non-imaging data curation.** Curation of clinical (non-imaging) data requires initial anonymization, which was also performed using the CM-Proxy from CMRAD (see previous section). The important subsequent step is data harmonization. After selecting the ICGC ARGO dictionary, we performed a systematic mapping of the parameters used to dictionary values. Then, for each use case, we created a table that gathers all the variables, as well as their respective accepted values. This table will be filled at each site semi-automatically (a script will be used to feed the table, and then the result will be checked manually). Finally, we will adapt quality control programs made by the ICGC ARGO team, to make sure that the variables that present dependencies are correctly linked.

Appendix 3 - INCISIVE

3.1 Architecture

The goal of INCISIVE is to build upon a federated storage approach. The architecture, shown in Figure 7, is subject to changes and updates in order to integrate all relevant user requirements and specifications.

The INCISIVE platform is divided in three parts:

1. The data preparation toolset: It is a set of tools that operate at each data provider's site before the data federation stage so that the data becomes GDPR-compliant and appropriately processed (e.g., annotated, where relevant). The main tools that support this

process are the data pseudo/anonymisation tool, the data annotation tool, the data curation tool and the data quality check tool. These tools altogether ensure that the data undergoes a correct pre-process to fulfil the system preconditions before being stored in the INCISIVE platform.

2. The federated space: It is the Cloud environment that contains the centralized services required to offer the federated INCISIVE functionalities.

3. The federated node: It is the node that is hosted by the data provider where the data is stored. The data does not leave the related premises; therefore, the data partners keep full control.

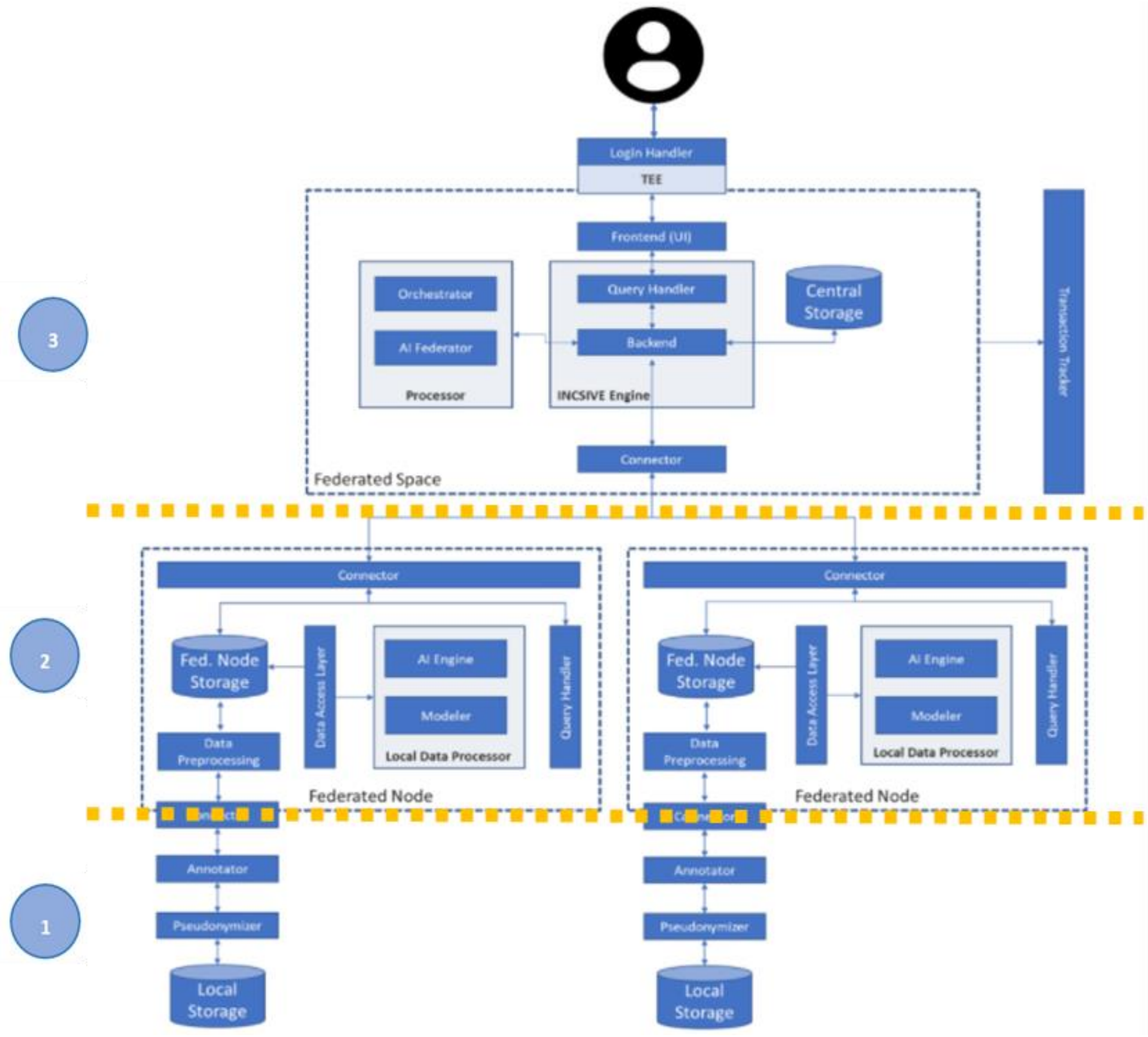

Figure 7. INCISIVE preliminary architecture.

The repository is an aggregation of data management layers that ensure the sharing and use of health data (including images) in an interoperable, secure and meaningful way. After using the INCISIVE data preparation tools for data curation, data de-identification/anonymization and annotation, the user uploads the data into the federated node (local data storage based on GDPR regulation). Then, the data is available through the Federated Repository mechanisms (data sharing according to FAIR principles and data management security based on GDPR) at the federated node so the user can perform queries over data and develop/ train/test AI models for cancer management and research with it thanks to a specific APIs (INCISIVE search engine and INCISIVE Federated learning).

3.2 Curation Process

Imaging data used in the INCISIVE project are in DICOM format. INCISIVE utilizes pseudonymization for retrospective imaging data used for model training, in order to provide sufficient data protection, without jeopardizing imaging data value. More specifically, INCISIVE aims to:

- Remove/obscure data fields that aren't required to fulfil the project's goal (using the data minimization concept).
- Hash (random value) most frequent indirect identifiers (such as birth date, examination time, and hospital).
- Replace the patient's name with a pseudonym, so that uploaded images may only be linked to the patient by the Data Provider who gave the data. In the same way, the Patient ID will be changed.
- Retain imaging information necessary for research purposes.

Apart from metadata stored in DICOM fields, personal information can be found ‘burned-in’ in image pixel data, which is especially common in some imaging modalities, such as ultrasound (Figure 8). Format varies depending on the device vendor, model, modality and software version, though the location is generally consistent. The presence of burned-in data is indicated in DICOM field (0028,0301), however its use is optional, and its absence implies uncertainty about whether the image contains burned-in annotations. INCISIVE will track and remove burned-in elements to ensure an efficient de-identification procedure.

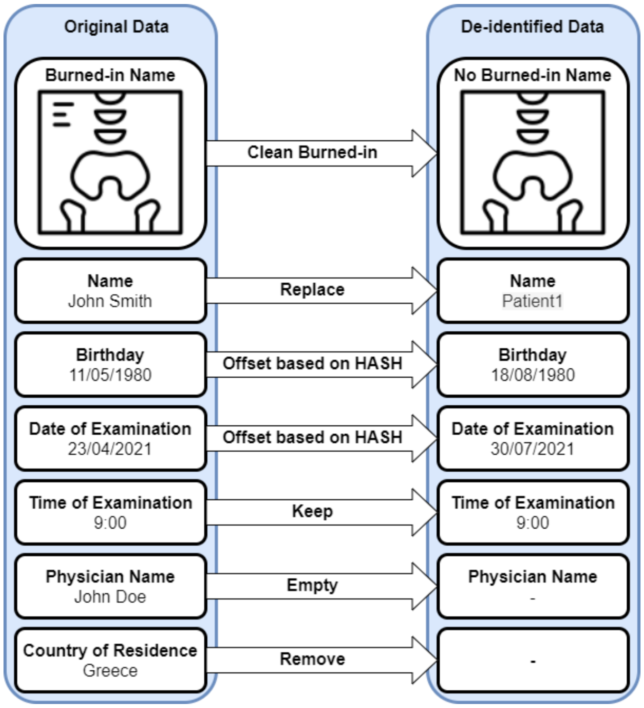

Figure 8. Illustration of DICOM metadata and burned-in annotations de-identification.

The De-Identification Process proceeds as follows:

Step 1: Pseudonymization Mapping: Data providers should be able to identify their data. Thus, they are instructed to manually and separately create a pseudonymization mapping table. Re-identification of data, once it has been pseudonymized, is only possible for a significant cause, and can only be performed by authorized staff of the original Data Provider.

Step 2: DICOM de-identification procedure: Before being securely transferred to the Temporary repository, each Data Provider de-identifies all provided data locally and independently. Data providers should use the recommended CTP DICOM Anonymizer de-identification tool. At submitting sites, the CTP DICOM Anonymizer utility is installed on a regular desktop computer, and Data Providers' workers are instructed on the method (e.g., dedicated workshop, provision of instructions on how to use the tool). To prevent eliminating any vital information, data providers should not use any other de-identification software prior to the INCISIVE system (e.g., possibly a PACS-integrated de-identification tool). If a Data Provider decides to use another de-identification tool instead of CTP DICOM Anonymizer, the tool must comply with the de-identification profile.

Step 3: Quality Control: The de-identification outcome will be evaluated on the premises of Data Providers. The data set may be uploaded to the data repository if the test results are regarded as sufficient in the context of the specified profile below. The method for de-identification during the upload of imaging data to the INCISIVE data infrastructure is represented in Figure 9, which illustrates the data pipeline that will be followed.

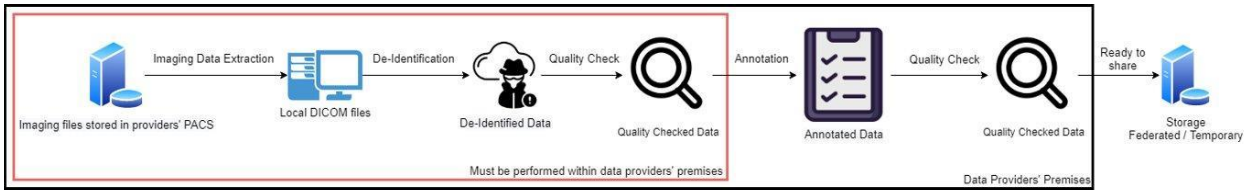

Figure 9. Data flow pipeline.

Annotation: The annotation tool was designed in two stages. During the first stage, the general user requirements were gathered, which were used to choose an existing open-source annotation tool. The external tool chosen was ITK-Snap and it fulfilled most of the data providers' needs during the initial stage of the annotation procedure of INCISIVE. It provided a manual, as well as minor semi-automatic segmentation functionalities. During the second stage, the specific user requirements of the INCISIVE partners were gathered, which were then used to implement the INCISIVE semi-automatic tool. The developed annotation tool provides semi-automatic annotation functionalities by utilizing pre-trained TensorflowJS models on the browser, thus achieving both high usability and privacy from the user perspective view.

After the de-identification is complete, the **annotation** procedure of INCISIVE is executed, as presented in Figure 9. The medical expert annotators are instructed to follow the annotation protocol of their organization, with respect to (a) the type of annotation (classification or segmentation), (b) the imaging modality and (c) the disease type in question. Moreover, visual checks by the medical annotators are performed to discard or fix images with burned-in annotations. Where feasible, the annotation process is repeated by more than one annotator to increase the quality of annotations.

Appendix 4 – ProCAncer-I

4.1 Architecture

The ProCAncer-I aims to deliver an infrastructure that follows the principles of open source, FAIR data access, common look-n-feel, common authentication and authorization, layered, developing of modelling service, modelling service certification and cloud infrastructure independence. The logical view of the ProCAncer-I platform with the main domain specific areas of functionality of the system is shown in Figure 10.

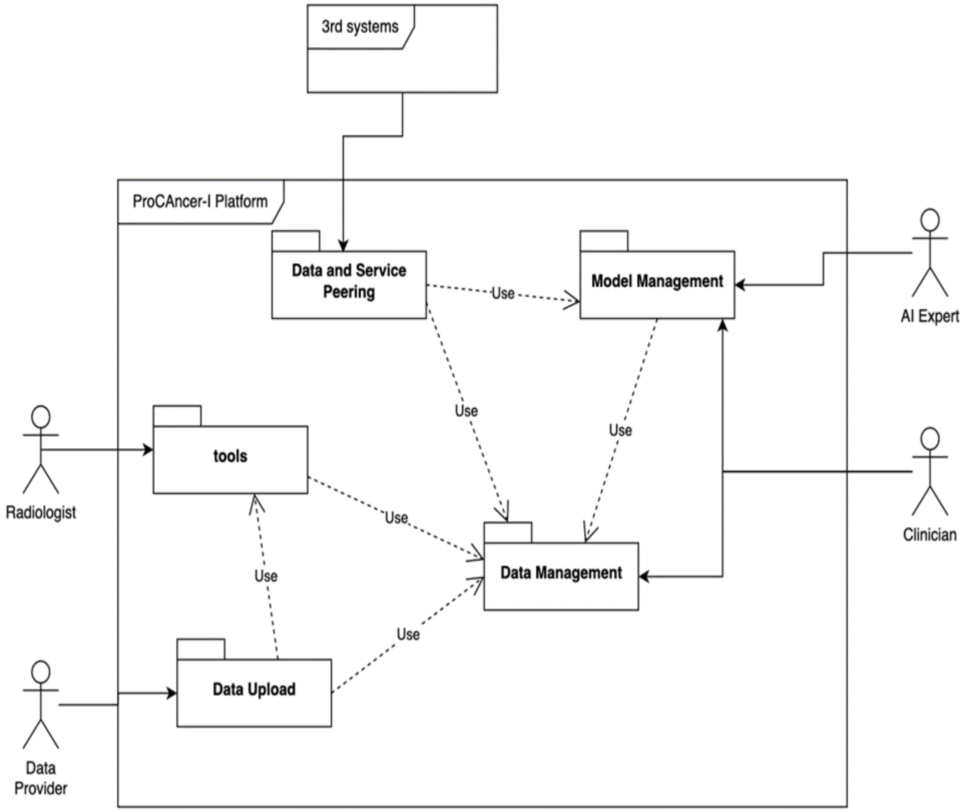

Figure 10. The main subsystems of the ProCAncer-I platform.

The following subsystems are identified:

- Data ingestion and upload [28]. This includes all the infrastructure (tools, services, cloud resources) that allows a data provider to upload their data sets according to the project's guidelines and best practices (e.g., anonymization) so that they become integrated to the curated cancer-related data managed by the system.

- Data Management, which supports the “data at rest” scenarios, is the core of the platform supporting all the other subsystems for the storage, efficient indexing, curation, and retrieval of the data.
- Domain specific tools, for example for image analysis and preprocessing, which support domain experts to annotate and curate the imaging data.
- Model management. This is part of the platform supporting the management of computational and AI tools and models. It allows searching for available models, the development of new ones, model execution and monitoring, etc.
- Data and Service “Peering” supports the exchange of data and services with other research infrastructures using well-defined FAIR-enabled APIs and applications like the “Honest Broker”.

At the “solution architecture” of the ProCAncer-I project the platform is deployed on a private cloud using “cloud native” technologies to support scalability, elasticity, and observability [29] (Figure 11). On the lower layer of the platform, cloud infrastructure services like Kubernetes, container registry, persistence volumes, etc. provide the resources to address the computation and data requirements of the project and support the rest of the platform. On the Data Management specialized data repositories like the DICOM Image Store and the Clinical Data Warehouse are responsible for storing the raw data uploaded. The Metadata Repository implements the data integration and harmonization methodology and it’s a “one-stop shop” for data search, dataset creation, and efficient OMOP-compliant data indexing and management. The model development and serving subsystem consists of a suite of Machine Learning Operations (MLOps) technologies and tools to support the feature engineering, experimentation, training, development, deployment, and monitoring of advanced AI/ML models. Finally, the end user interface layer comprises the tools and applications that clinicians, researchers, modellers, and public users use in order to interact with the platform. In this layer, data ingestion and upload, data management and domain specific tools, e.g., for image segmentation, motion correction and co-registration, have been developed and the integration is being concluded to allow data providers to make their data sets available in the ProCAncer-I community securely and with full annotation. The remaining subsystems regarding model management and data and service peering are still being analyzed.

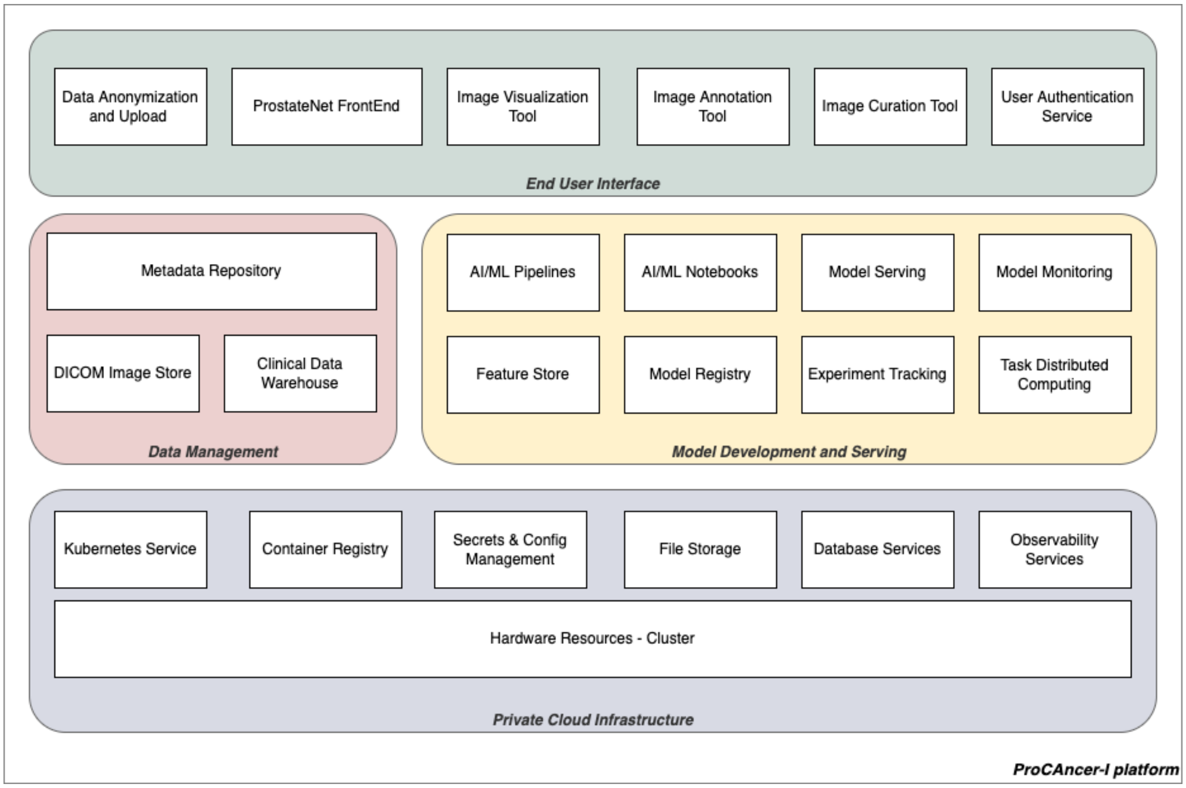

Figure 11. The main subsystems of the ProCAncer-I platform.

4.2 Curation Process

The ProCAncer-I platform collects and manages large amounts of multimodal data and metadata to enable the training of advanced AI models in an efficient and clinically oriented fashion for prostate cancer management. The ProCAncer-I platform storage, ProstateNet, is comprised of 3 components, the DICOM Object Store which stores medical imaging data, the Clinical Data Document Store which stores the clinical data, and the Meta-data Catalog which stores metadata and semantic annotations to enable rich search and discovery of data and its exploitation. The flow of data is illustrated in Figure 12. The clinical partners use a local, integrated eCRF and data upload tool to organize the DICOM studies and complete the clinical information, validate the use case, anonymize data and upload data to the cloud staging area. Each Clinical Partner has its staging area where its users are able to run the data curation tools, verify the anonymization and completeness of data, and submit validated cases to the ProstateNet repository.

On top of the available data, multiple curation tools are available, guiding the users through a set of steps for the curation: motion-correction, co-registration, and quality check, and final approval and storage of the derived images or their rejection, after the manual inspection of the results.

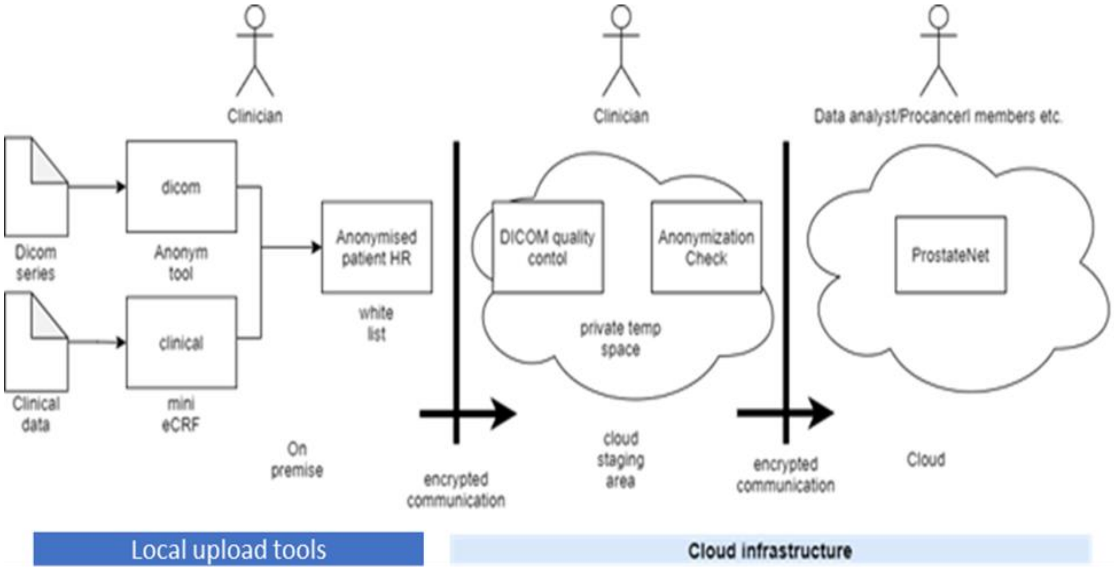

Figure 12. Data acquisition flow.

Initially, a study can be either identified by its modified/anonymized Study Instance UID and uploaded via a DICOMweb RESTful API or uploaded as a ZIP/TAR file to the server. In the pre-processing stage, the curation tool attempts to identify all included studies, in case multiple studies are present, especially in a manual upload, and group the DICOM series per study. Afterwards, certain public DICOM tags, such as the Series Instance UID and Series Description, alongside additional metadata, like the image's shape, zooms, and the plane of acquisition, are extracted to serve as indexing keys, user-facing information, and inputs to upcoming processing steps. During this stage, the tool also attempts to discover a default static series to be used in the image co-registration phase. The default static series must be (a) 3D, (b) T2w (based on the series description and/or protocol name), (c) axially acquired (based on the image's zooms extracted from the affine matrix), and (d) not the result of a curation function. At the end of this pre-processing phase, a series of asynchronous tasks are scheduled to preemptively compute curation outcomes with default parameters.

The motion-correction application performs inter-volume motion-correction of a DWI or DCE series by computing an affine transformation to register two 3D volumes. The first volume is automatically selected as the reference volume. The selection of the reference volume, as well as the exclusion of slices with high levels of motion, could be made configurable by the user in

future versions. The motion-corrected series can be concurrently reviewed for intra- and inter-volume motion in two side-by-side viewers.

The co-registration application co-registers the motion-corrected series to a T2w image. The moving (motion-corrected) and static (T2) series are also colour-coded. If the result of the co-registration is unsatisfactory, the registration hyperparameters may be refined in order to re-run the process.

After the curation of the study has finished, each new image is uploaded to the Cloud Staging Area as a new DICOM series with a freshly generated Series Instance UID and any related series (of the original study) added to the Referenced Series Sequence public DICOM tag and curation-related information is added to the Metadata Catalogue.

Data Annotation. For the annotation of DICOM studies, an annotation tool environment has been developed and is ready to be integrated with the rest of the components of the ProCancer-I system. This tool has been designed to follow a DICOM in – DICOM out approach. As a main feature, it provides the ability to draw regions of interest (ROI) on the medical image by using a brush. The brush allows the user to easily segment regions of interest in the image by marking the desired pixels.

Appendix 5 – PRIMAGE

5.1 Architecture

The PRIMAGE platform consists of two main components:

- The web interface, which is used for data ingestion and is the basis to build the clinical decision support system (CDSS) allowing for the visualization of the patient information (imaging data together with clinical and molecular information), its analysis and the exploration of the results using advanced visualization tools.
- The HPC cloud infrastructure, is being used for all computationally intensive tasks and AI research.

Figure 13 illustrates the main components of the web interface and their connection with the HPC cloud infrastructure, based on Quibim Precision® platform. It consists of three main layers:

- Front-end: this layer is exposed to the end user to interact with the software. Users can access the user interface through (https://primage.quibim.com/)

- Back-end: this layer is built by different services that process requests from the user interface or external applications, allows batch image data ingestion by connecting the platform to the PACS in the cloud or schedules the execution of analysis pipelines integrated in the platform (using Azure Kubernetes Service, AKS).
- Persistence layer: this layer is used to persist all the non-volatile information. It is composed of the PACS in the cloud, based on dcm4chee, that serves as a gateway to upload imaging studies in batches; MongoDB, a NoSQL database running on Mongo Atlas, to store all the non-imaging information in the platform database, this information is accessed through the platform REST API; and the cloud storage, which persists all the files (imaging studies, results, etc.) in Microsoft Azure. The cloud storage is synchronized with the HPC cloud infrastructure storage using OneData.

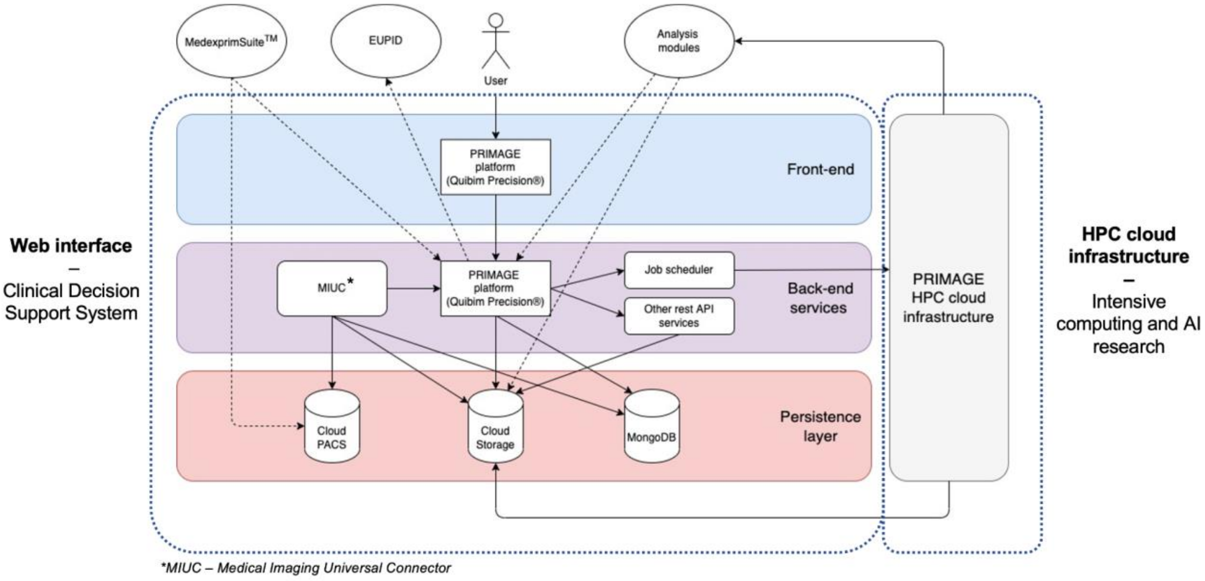

Figure 13. PRIMAGE web platform architecture and connection with HPC cloud infrastructure.

5.2 Curation Process

PRIMAGE has built a database of hundreds of cases from different data sources, including European clinical data registries (i.e., the European Neuroblastoma Registry from the SIOPEN-r-net), local registries from the clinical partners of the project, and external collaborators. Therefore, according to the needs of each institution, different data uploading processes have been agreed and designed to allow the integration of all the data in the PRIMAGE platform. For

the project, clinical, molecular and imaging data are being collected. To homogenize the clinical and molecular data, independent eCRFs have been designed and integrated in the platform for NB and DIPG. These eCRF have been designed to guarantee data validity, integrity and completeness. Within the eCRF, quality checks are incorporated to guarantee the quality of the data, therefore, they cannot be signed unless the information is within some predefined ranges, with the appropriate chronological order for the dates and with all the mandatory fields fulfilled.

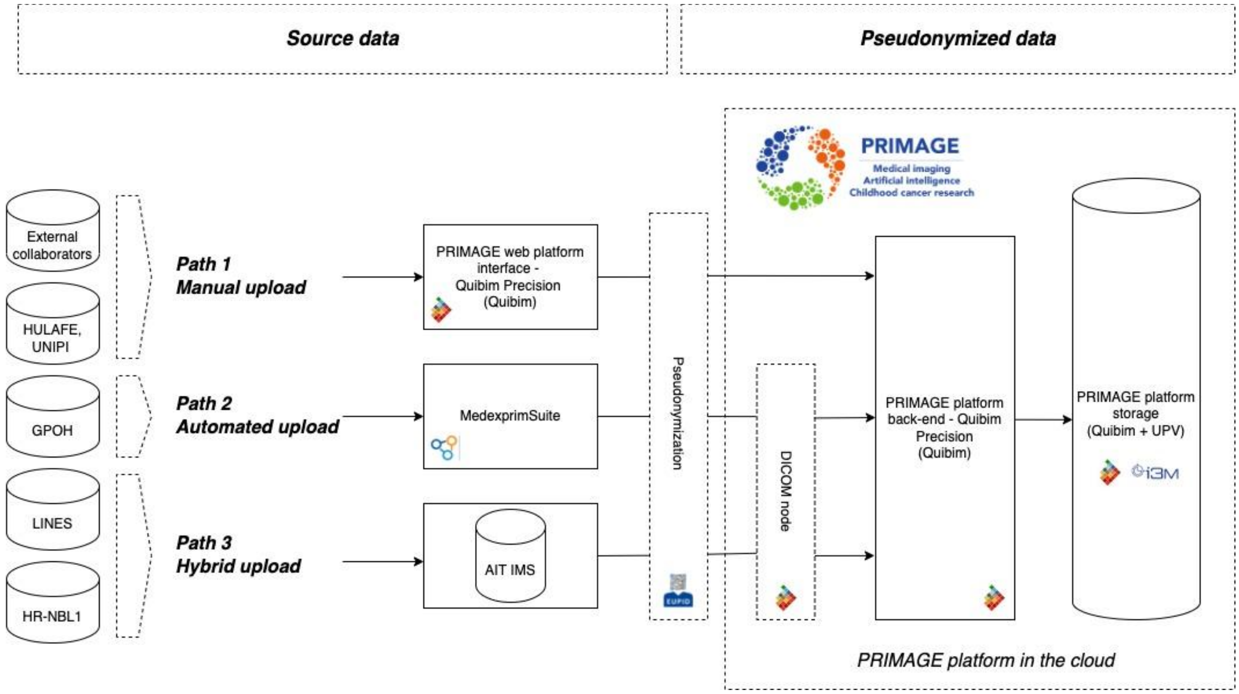

Figure 14. Data flow for imaging data upload to the PRIMAGE central repository.

Figure 14 shows three different paths used for data uploading:

- Manual upload: the PRIMAGE web platform, through the user interface, allows the uploading of both imaging and clinical and molecular data on a per patient basis.
- Automated upload: MedexprimSuite, integrated directly in the local sites, performs the automated upload of the data to the platform.
- Hybrid upload: the data from the SIOPEN-r-net registries was already collected and centralized. Therefore, to access this data, first, the data is downloaded manually from the central repository and, through batch upload, sent back to the PRIMAGE platform.

Once the imaging studies are uploaded to the platform, an MR series classifier is used with the objective of automating the process of curation and post-processing of MR images. It consists of a tool that provides standardized labelling (e.g., T2W, T1W, FLAIR, DWI, DCE) for each MR sequence.

A tool was developed to assess the quality of images and provide a quality score on images. Based on the score, studies may either be automatically approved, excluded, or quarantined for manual check. To do so, 6 image metrics were extracted: 3 of them related to the signal SNR (signal-to-noise ratio), CNR (Contrast to noise ratio), VAR (Variance); and 3 aimed for artefact detection FBER (Foreground-background energy ratio), CJV (Coefficient of variation), EFC (Entropy Focus criterion. It is important that the inference is based on the extraction of metrics, to have a better understanding of which aspect of the image the lack of quality is due to. For those metrics to be calculated, the foreground was separated from the background using a method based on the Otsu threshold and morphothematic. Finally, a dataset with images labelled as "good" or "bad" by a radiologist was used to train a classification model, which was then used to obtain the final quality score.

The uploaded and labelled imaging studies can then be annotated by means of delineations of regions of interest. For the project, the primary tumour is being segmented from a small batch of patients to allow the training of Deep Learning models for automatic tumour segmentation. This annotation can be conducted using the PRIMAGE platform user interface, where a zero-footprint DICOM viewer is integrated with different annotation tools. However, already existing segmentation files stored in NIfTI or DICOM RT Struct format can also be uploaded to the platform.

Finally, to reduce noise and variability across imaging studies, different tools for imaging preprocessing have been developed, validated and integrated into the platform. These tools include image denoising [30], motion correction and image registration.

All these preprocessing algorithms generate new DICOM series, associated with the original study, that are stored in the PRIMAGE database for their use as input images in other analyses.
